# Investigation of genetic and lifestyle risk factors associated with Epstein-Barr virus reactivation in the Thai population

**DOI:** 10.3892/br.2026.2123

**Published:** 2026-02-20

**Authors:** Janpen Bamrungthai, Tipaya Ekalaksananan, Sutida Pongpakdeesakul, Theeradon Rueankaew, Wanpiya Fhakdee, Darin Duangchai, Fernladda Kattiwong, Pornsiri Lanpol, Sasiwimon Sumala, Kachain Chantawong, Sureewan Duangjit, Sureewan Bumrungthai, Chamsai Pientong

**Affiliations:** 1 Department of Otorhinolaryngology, Sunpasitthiprasong Hospital, Maung Ubon Ratchathani, Ubon Ratchathani 34000, Thailand; 2 Department of Microbiology, Faculty of Medicine, Khon Kaen University, Mueang Khon Kaen, Khon Kaen 40002, Thailand; 3 Division of Microbiology and Parasitology, School of Medical Sciences, University of Phayao, Maung Phayao, Phayao 56000, Thailand; 4 Division of Biopharmacy, Faculty of Pharmaceutical Sciences, Ubon Ratchathani University, Maung Ubon Ratchathani, Ubon Ratchathani 34190, Thailand; 5 Division of Pharmaceutical Chemistry and Technology, Faculty of Pharmaceutical Sciences, Ubon Ratchathani University, Maung Ubon Ratchathani, Ubon Ratchathani 34190, Thailand; 6 Division of Biopharmacy, Faculty of Pharmaceutical Sciences, Ubon Ratchathani University, Maung Ubon Ratchathani, Ubon Ratchathani 34190, Thailand

**Keywords:** Epstein-Barr virus, head and neck cancer, *TNF-α*, Thailand

## Abstract

Current data on the genetic and lifestyle factors associated with Epstein-Barr virus (EBV) reactivation in the oral cavity are limited for the Thai population. Furthermore, comprehensive data linking EBV reactivation to head and neck cancers in Thailand remains scarce. The present study aimed to detect EBV reactivation using quantitative PCR in normal oral buccal cells and to examine the associated risk factors. A total of 982 oral buccal cell samples collected across Thailand were analyzed. EBV was detected in 36% (350/974) of samples when targeting the Epstein-Barr nuclear antigen-1 gene, 52% (458/885) of samples when targeting latent membrane protein-1 (*LMP-1*) and 20% (196/981) of samples when both genes were investigated. The highest prevalence of *LMP-1* and dual gene positivity was observed in individuals aged 11-20 years. Several SNPs in the *TNF-α* promoter region, including rs1452146766, rs1799964, rs1554283139, rs924800313, rs1799724 and rs1771099055, were more frequently observed in EBV-positive samples than in EBV-negative samples. Notably, the *TNF-α* mutation rs1799964 (-1031 TC and CC) was present in 17.3% vs. 9.3% of EBV-positive cases, respectively. Multivariate analysis identified sex, smoking, alcohol consumption, soft drink intake, age of 21-30 years and having four children as significant factors associated with EBV reactivation. In the 21-30-year-old age group, *LMP-1* positivity was elevated, and higher rates of alcohol use, sexual activity and oral ulcers were observed. Furthermore, in individuals with mouth ulcers, the *TNF-α* mutation (TC; n=81) was more common than wild-type TNF-α (CC; n=16).

## Introduction

Head and neck cancers, including cancers of the pharynx, larynx, nasopharynx, oropharynx, salivary glands, oral cavity, nasal cavity and external auricle, rank as the seventh most common group of cancers worldwide (1). Although tobacco and alcohol use are well-known risk factors, Epstein-Barr virus (EBV) infection has also been implicated in numerous cases (1-3).

Among these cancers, oral cavity cancer is the most prevalent, with 377,713 new cases reported globally in 2020. The global age-standardized incidence rate (ASR) for oral cavity cancer was 6.0 for men and 2.3 for women (4). In the same year, 133,354 cases of nasopharynx cancer, 98,412 cases of oropharynx cancer, 84,254 cases of hypopharynx cancer, 184,615 cases of larynx cancer and 53,583 cases of salivary gland cancer were reported globally (4). In Thailand, oral cavity cancer has consistently ranked among the most common malignancies in men, as demonstrated by population-based cancer registry data, multicenter studies, and national epidemiological analyses spanning from the 1990s to recent years (5-11).

Globally, the incidence of early-onset cancer in young adults increased by 79.1% from 1990 to 2019, with a projected increase of 31% by 2030 (12,13). A similar trend was observed for oral cavity and pharyngeal cancers among young adult women (12). Adolescents and young adults are defined as individuals aged 15-49 years (12-14).

The causes behind the rising cancer rates in younger populations, whether genetic or environmental/lifestyle-related, remain unclear. Proposed hypotheses include chronic inflammation, smaller family sizes, gut microbiome alterations, processed food consumption, microplastic exposure, sedentary lifestyles, family history of cancer, early screenings, germline mutations and other genetic alterations (15,16). Understanding potential risk factors, including EBV reactivation, may support early prevention strategies for head and neck cancer (3). EBV may also participate in infection events with other viruses, including human papillomavirus, BK polyomavirus, human cytomegalovirus and herpes simplex virus (3). Currently, there is no screening biomarker for head and neck cancer in Thailand. Thus, head and neck cancer is only detected after the patient exhibits signs and symptoms. Furthermore, cases of oncogenic EBV infections in head and neck cancer are limited in Thailand (5-11).

Previous reports have suggested that EBV infection may promote the progression of oral squamous cell carcinoma (OSCC) (17,18). A meta-analysis of 13 case-control studies (686 patients with OSCC and 433 controls) confirmed a statistically significant association between EBV infection and increased OSCC risk (19). Furthermore, a study involving 315 Thai participants detected EBV infection in 27.2% of normal oral exfoliated cells and 72% of oral cancer cases (20). EBV is transmitted through saliva and genital secretions (21). Mekmullica *et al* (22) reported that EBV antibody-positive status in the Thai population was associated with low family income (≤10,000 baht/month) and age >1 year. Pongpakdeesakul *et al* (23) identified alcohol consumption, second-hand smoke and using tap water for brushing teeth as risk factors for EBV DNA reactivation in blood samples.

EBV is separated into the α, β and γ subfamilies. Nearly 95% of the population is infected with EBV throughout life (24). EBV comprises linear double-stranded DNA, ~172 kb in length. EBV contains at least 11 genes spanning EBV-encoded RNAs (EBER-1 and EBER-2), EBV nuclear antigens (EBNA-1-6) and latent membrane proteins (LMP-1, and LMP-2A and -2B). The latent phase of EBV infection is associated with numerous types of cancer, such as lymphoma, Hodgkin lymphoma and nasopharyngeal carcinoma, due to the expression of EBERs, EBNA-1 and the three LMPs, among others (25,26). PCR could be used to detect EBV reactivation in blood or saliva. DNA-positive results indicate that the virus is actively replicating (27-29).

EBV and TNF-α interact in a complex, context-dependent manner, with TNF-α functioning as both a tumor promoter and a cancer inhibitor depending on EBV activity. TNF-α can inhibit the lytic replication of EBV by reducing the expression of viral proteins such as *Bam*HI Z Epstein-Barr virus replication activator and R transactivator. TNF-α affects the glutathione peroxidase 4 protein and can inhibit EBV reactivation. Furthermore, EBV-infected T cells exhibit increased secretion of TNF-α, potentially promoting cancer development (30-32). *Bam*HI Z fragment leftward open reading frame 1 (BZLF-1) also suppresses TNF-α production to facilitate viral propagation, while LMP-1 downregulates TNF-α receptor 1, thereby preventing apoptosis and promoting proliferation (30-32).

Currently, updated data on EBV reactivation risk factors in the Thai population are limited and mostly focus on antibody detection (5-11), and evidence linking genetic mutations to EBV infection is also limited. Therefore, the present study aimed to detect EBV reactivation using quantitative PCR (qPCR) and to investigate associated risk factors in normal oral buccal cells using logistic regression.

## Materials and methods

### Specimens

A total of 982 human oral buccal DNA samples were collected from donors across Thailand (samples collected from the village population of Ubon Ratchathani and Phayao province) between December 2016 and March 2022, as reported in previous studies (33,34), and analyzed using qPCR as described subsequently. Participant age ranged between 3 and 90 years. Individuals who were easy to contact or reach were included. Individuals living in Thailand were included, and there were no other inclusion criteria. Individuals who could not perform oral swirling with PBS and patients with cancer were excluded. Among the samples, 599 included information on sex, health status and life history (such as presence of mouth ulcers, congenital diseases, family history of cancer, soft drink consumption, alcohol use and smoking). This information was used for risk factor assessment via regression analysis. Percentages based on demographic data (599 samples) were used to generate a surface chart (contour), with patients divided into various age groups.

All variable factors (such as presence of mouth ulcers, congenital diseases, family history of cancer, soft drink consumption, alcohol use and smoking) were used for univariate regression model analysis, and only significant variables (P<0.05) were used for the multivariate regression model analysis.

The samples used in these experiments were collected between 2016 and 2022, and leftover samples were used. Therefore, some DNA samples were depleted or unavailable. The number of lost samples was ~30 samples.

The required sample size was calculated using the following formula: N=Z^2^_1-a_ P(1-P)/d^2^ (35), where EBV prevalence (P) ranged between 3.8 and 33.75%, Z=1.96 for a 95% confidence level, and d=0.01 or 0.05, resulting in a sample size range of 56-1,404. The study was approved by the Human Ethics Committee Ubon Ratchathani University (Ubon Ratchathani, Thailand; approval no. UBU-REC-68/2567; valid from March 20, 2024, to March 19, 2026). All procedures followed The Declaration of Helsinki (36), Belmont Report (37), Council for International Organizations of Medical Sciences guidelines (38) and the International Conference on Harmonization in Good Clinical Practice standards (39). Written informed consent was obtained from all participants or their legal guardians (for patients <18 years old).

### DNA extraction

Oral buccal cells were collected 1 h after tooth brushing using 10 ml sterile 1X PBS. Mouthwash samples were centrifuged at 8,000 x g for 5 min at room temperature and resuspended in 500 *µ*l lysis buffer (10 mM Tris-HCl, pH 7.8; 5 mM EDTA; 0.5% SDS) with 25 *µ*l proteinase K (20 mg/ml stock) (40). After protein precipitation using 400 µl of 5 M potassium acetate, the supernatant was mixed with an equal volume of isopropanol. DNA was pelleted by centrifugation at 10,000 x g for 10 min at 4*˚*C, and washed with 70% ethanol. The supernatant was removed, and the DNA was resuspended in Tris-EDTA buffer (10 mM Tris, pH 7.8; 1 mM EDTA), and then stored at -20˚C (34).

### EBV DNA detection via qPCR

EBV DNA (targeting *EBNA-1* and *LMP-1* genes) was detected using qPCR with specific primer sets (17,41). For *EBNA-1*, the following primers were used: Forward (primer name, QP3), 5'-CCACAATGTCGTCTTACACC-3' and reverse (primer name, QP4), 5'-ATAACAGACAATGGACTCCCT-3' (41). For *LMP-1*, the following primers were used: Forward, 5'-CAGTCAGGCAAGCCTATG-3' and reverse, 5'-CTGCTTCCGGTGGAGATG-3'. The expected PCR product sizes were 99 bp for *EBNA-1* and 106 bp for *LMP-1* (17). The B95 cell line was used as a positive control for EBV DNA detection. The B95-8 cell line sequence was identified according to the V01555.2 EBV genome from the National Center for Biotechnology Information (NCBI), and authenticated prior to experimentation. B95 cells were cultured in RPMI medium (Lonza Group, Ltd.) supplemented with 10% FBS (HyClone; Cytiva), 100 U/ml penicillin G, 100 µg/ml streptomycin sulfate and 2 mM glutamine at 37˚C with 5% CO_2_ (42).

EBV genome, strain B95-8 profiling was performed by Professor Jaap M. Middeldorp (Department of Pathology, VU University Medical Center, Amsterdam, The Netherlands), and the profile matched the reference profile available from NCBI (43).

The *EBNA-1* and *LMP-1* primers were validated in previous studies by Stevens *et al* (41) and Heawchaiyaphum *et al* (17). *EBNA-1* and *LMP-1* primers were detected in duplicate.

Reactions were prepared using 5X FiREPOL Eva Green qPCR Mix Plus (Solis BioDyne), with a final composition consisting of 1X FiREPOL master mix, 0.4 pM of each primer (forward and reverse), 2 µl DNA template and distilled water (DW) to a final volume of 20 µl. The qPCR conditions were as follows: Initial activation at 95˚C for 12 min, 40 cycles of denaturation at 95˚C for 15 sec and annealing/elongation at 60˚C for 30 sec, and melting at 65-95˚C (0.5˚C/5 sec/step).

### Conventional PCR

Conventional PCR was performed as described previously (44). Detection of the *TNF-α* promoter (rs1799964; -1031 T>C) was performed using allele-specific PCR. For the T allele, the following primers were used: Forward, 5'-AAGGCTCTGAAAGCCAGCTG-3' and reverse, 5'-CCAGACCCTGACTTTTCCTTCA-3'. For the C allele, the following primers were used: Forward, 5'-GAAGCAAAGGAGAAGCTGAGAAGAC-3' and reverse, 5'-CTTCCATAGCCCTGGACATTCT-3'.

The expected PCR product sizes were 444 and 316 bp for the TT genotype, 444, 316 and 174 bp for the TC genotype, and 444 and 174 bp for the CC genotype (44).

To assess the long region of the *TNF-α* promoter, 20-25 randomly selected EBV-positive and EBV-negative samples from a previous study were used (23). The primers, which were previously designed and original in-house primers for amplification of the long promoter region, were as follows: Forward, 5'-AGCTGTGGGGAGAACAAAAGG-3' and reverse, 5'-GAGGGCGGGGAAAGAATCAT-3'. The PCR product size was 1,102 bp.

Reactions were prepared using a 5X FiREPOL Ready-to-Load Master Mix (Solis BioDyne) with the following components: 1X FiREPOL master mix, 0.4 pM of each primer (forward and reverse), 3 µl DNA template and DW to a final volume of 25 µl. The PCR conditions were as follows: Initial activation at 95˚C for 5 min, 40 cycles of denaturation at 95˚C for 1 min, annealing at 58˚C for 1 min and elongation at 72˚C for 1 min, followed by a final elongation step at 72˚C for 5 min. PCR products were analyzed by 2% agarose gel electrophoresis in 1X Tris-acetate-EDTA buffer at 100 V for 40 min.

### DNA sequencing

Sanger sequencing was performed to analyze the 1,102-bp region of the *TNF-α* promoter (long region), covering genomic region NC_000006.12 (positions 31574417-31575499). The aforementioned 20-25 randomly selected samples were sequenced to verify both EBV-positive and EBV-negative cases. Sequence data were analyzed using BioEdit (version 7.2; https://bioedit.software.informer.com/7.2/), a biological sequence alignment editor, and compared against the GenBank reference sequence (region, NC_000006.12; positions, 31574417-31575499; https://www.ncbi.nlm.nih.gov/nuccore/NC_000006.12).

### Statistical analysis

Statistical analysis was conducted using IBM SPSS software version 16 (SPSS, Inc.). Data are presented as n (%). Pearson's χ^2^ test was applied to compare categorical variables between groups. For qPCR, each sample was analyzed in duplicate (technical replicates). Both univariate and multivariate logistic regression analyses were performed to evaluate associations [P-value, odds ratio (OR) and 95% CI]. P≤0.05 was considered to indicate a statistically significant difference.

## Results

### EBV DNA detection via qPCR and risk factors

Oral buccal cells were collected from 982 donors in Thailand, including 301 male (30.7%) and 681 female (69.3%) patients aged 3-90 years (mean age, 45.39±14.99 years). qPCR detected EBV-positivity in 350 out of 974 individuals (36%) based on the *EBNA-1* gene, and in 458 out of 885 individuals (52%) based on the *LMP-1* gene. Co-positivity for both *EBNA-1* and *LMP-1* was observed in 196 out of 981 individuals (20%) (Table I). DNA from the B95-8 cell line was used as a positive control.

There was a significant association between *EBNA-1* positivity and age. In particular, individuals aged 3-10 years had a higher prevalence of *EBNA-1* positivity (P<0.001) than other groups, while those aged 11-20 years showed a higher prevalence of *LMP-1* and dual gene positivity (P<0.001) than other groups (Table I).

**Figure 1:**
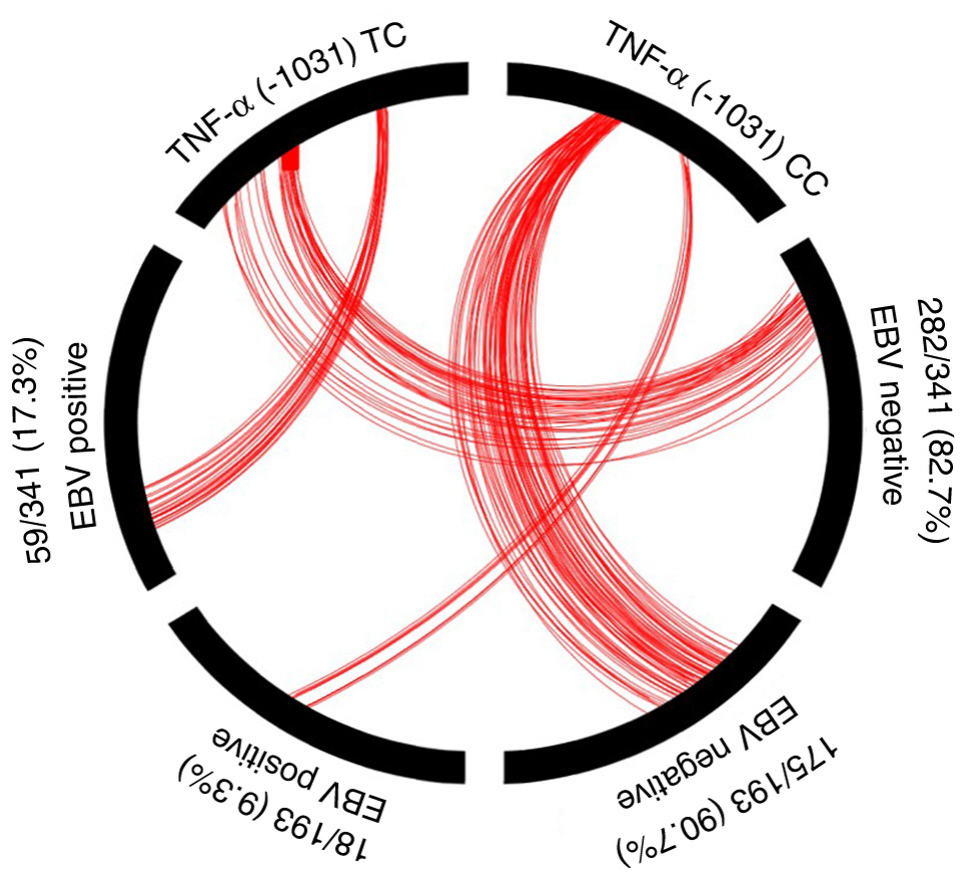
Association between *TNF-α* (-1031 TC) and dual gene positivity (both positive *EBNA-1* and *LMP-1*). *EBNA-1*, Epstein-Barr nuclear antigen-1; EBV, Epstein-Barr virus; *LMP-1*, latent membrane protein-1.

Using *LMP-1* gene detection, EBV positivity was significantly more prevalent in female patients (55%) compared with male patients (43%) (P=0.002; OR, 1.579; 95% CI, 1.185-2.105). In the *LMP-1* positive group, the *TNF-α* genotype *TC* (42%) was more common than the *TNF-α* genotype *CC* (31%) (P=0.014; OR, 1.601; 95% CI, 1.100-2.331). Additionally, individuals positive for both *EBNA-1* and *LMP-*1 had higher frequencies of the *TC* genotype (17%) than the *CC* genotype (9%) (P=0.012; OR, 2.034; 95% CI, 1.161-3.563) (Table I; Fig. 1). EBV status by age, sex and *TNF-α* status is shown in Table I. The association between *TNF-α* (-1031 TC) and dual gene positivity (*EBNA-1*- and *LMP-1*-positive) is shown in Fig. 1.

Results for the 599 buccal samples are presented in Table II. The proportion of samples positive for both *EBNA-1* and *LMP-1* was 13%.

For the 599 buccal samples with available demographic and lifestyle data (including sex, age, number of children, number of sexual partners, sexual activity, congenital disease, family history of cancer, betel nut chewing, mouth ulcers, alcohol use, smoking and beverage consumption), univariate and multivariate regression analyses were conducted.

Univariate analysis revealed that mouth ulcers, sex, hot tea consumption and sexual activity were significantly associated with EBV positivity (Table SI).

Univariate analysis revealed significant associations between *EBNA-1* positivity and several factors, including mouth ulcers, soft drink consumption, and the age groups of 3-10, 11-20, 21-30 and 41-50 years (P<0.05; Table SI). Being in the 11-20-year-old age group, alcohol consumption, smoking status, sexual activity and hot tea consumption were significantly associated with *LMP-1* positivity (P<0.05; Table SI). Notably, both alcohol consumption and smoking status were significantly associated with positivity for both EBV genes (P<0.05). Mouth ulcers were significantly associated with *EBNA-1* gene positivity based on univariate analysis (P=0.014; OR, 0.952) (Table SI).

**Figure 2:**
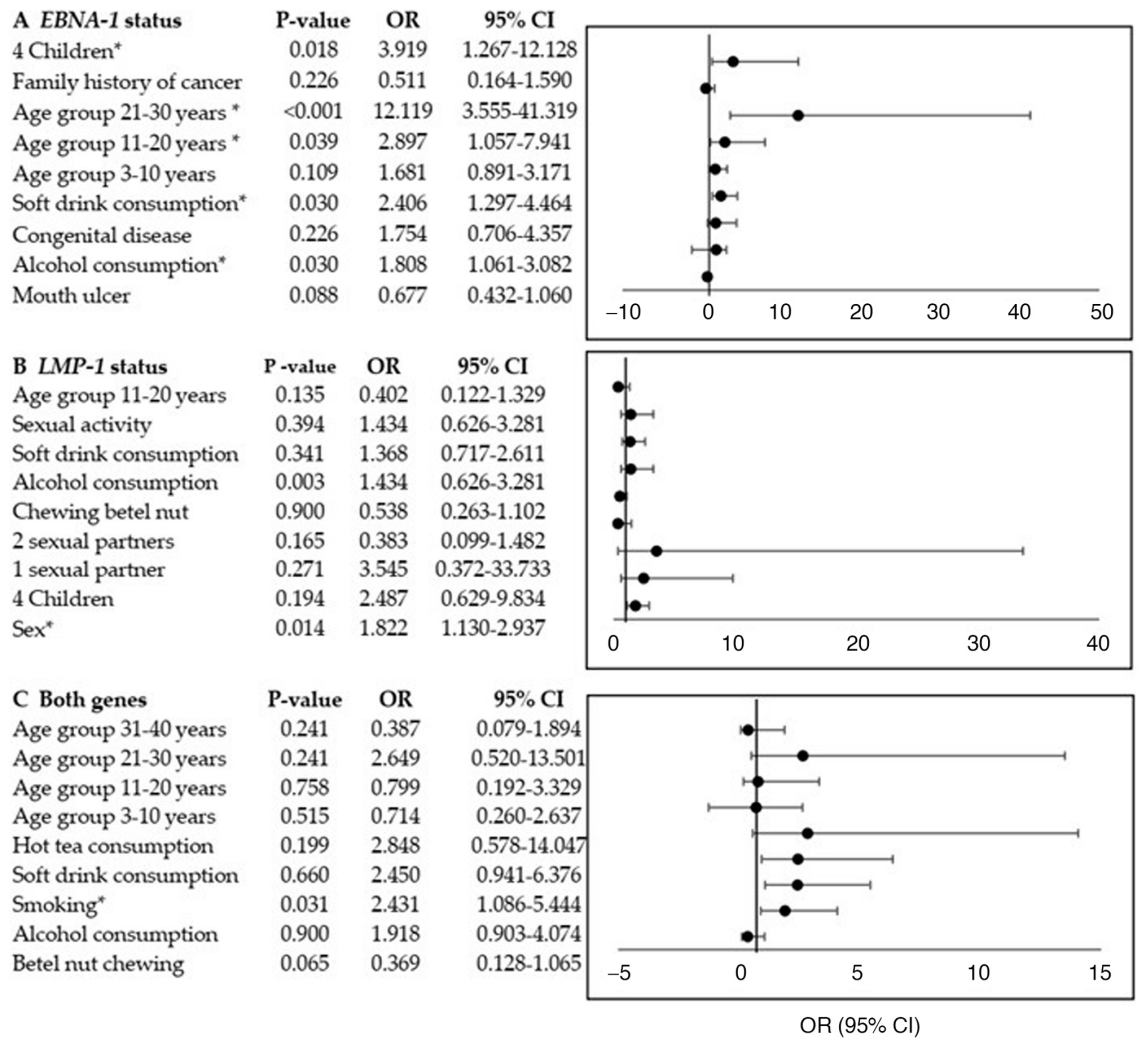
Multivariate binary logistic regression models showing associations between Epstein-Barr virus reactivation (*EBNA-1* and/or *LMP-1*) and risk factors. The x-axis represents ORs and the y-axis lists risk factors. (A) *EBNA-1* status, (B) *LMP-1* status and (C) dual gene positivity. ^*^Statistically significant. *EBNA-1*, Epstein-Barr nuclear antigen-1; *LMP-1*, latent membrane protein-1; OR, odds ratio.

Multivariate analysis indicated that *EBNA-1* positivity was significantly associated with alcohol and soft drink consumption, being in the 11-20-year-old age group, being in the 21-30 year-old age group, and having four children. *LMP-*1 positivity was associated with sex, while smoking status was significantly associated with dual gene positivity (Fig. 2).

Overall, significant risk factors for EBV positivity included smoking, sex, soft drink consumption and age of 21-30 years.

**Figure 3:**
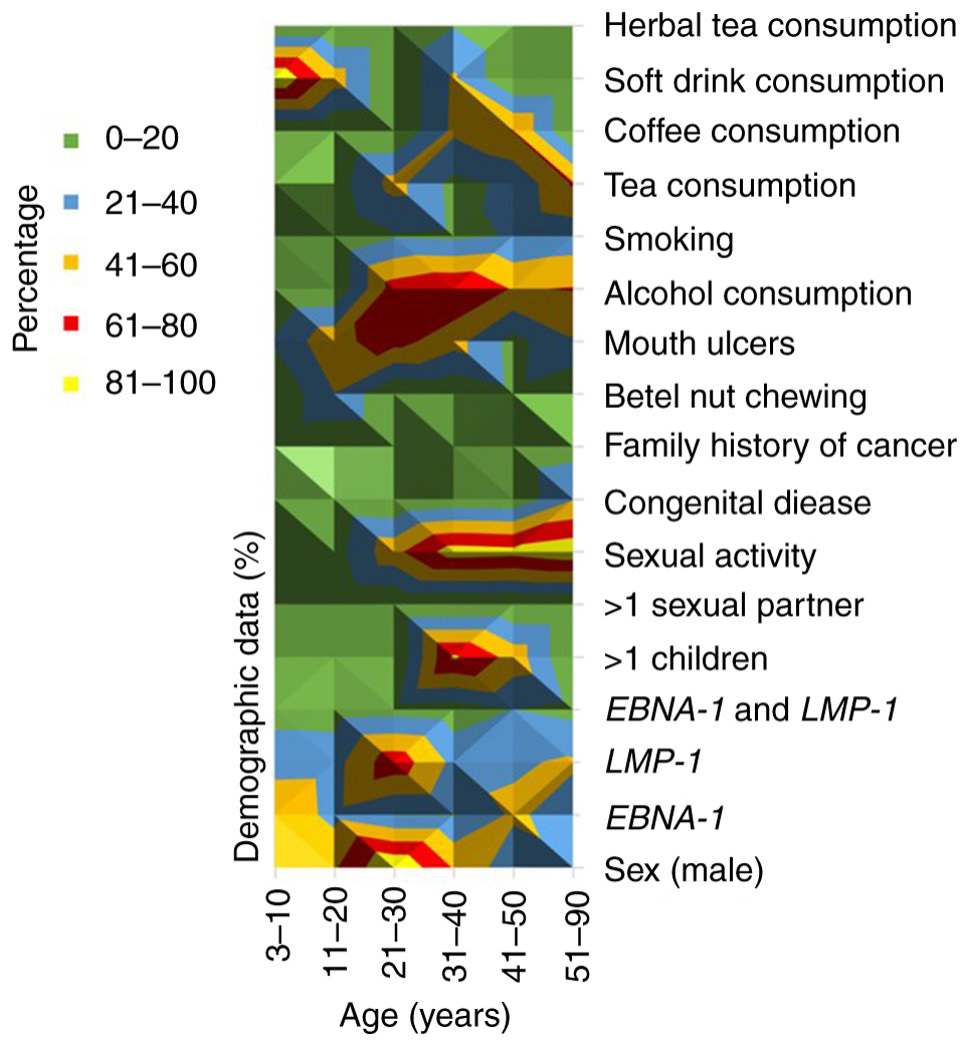
Exposure to risk factors of oral cancer across age groups. Colors represent the range percentage of exposure to environmental risk factors: 0-20%, green; 21-40%, blue; 41-60%, orange; 61-80%, red; 81-100%, yellow. Lighter colors and darker colors indicate lower and higher percentages, respectively (Table SII). Factors included Epstein-Barr virus status, sex, congenital disease, mouth ulcers, family history of cancer, smoking and sexual activity. *EBNA-1*, Epstein-Barr nuclear antigen-1; *LMP-1*, latent membrane protein-1.

Individuals aged 21-30 years showed high *LMP-1* gene positivity. Individuals aged 31-40 had a high level of alcohol consumption (79%), and individuals aged 31-40, 41-50 and 51-90 had a high level of sexual activity (90, 87 and 95%, respectively) (Fig. 3; Tables II and SII).

### DNA sequencing

**Figure 4:**
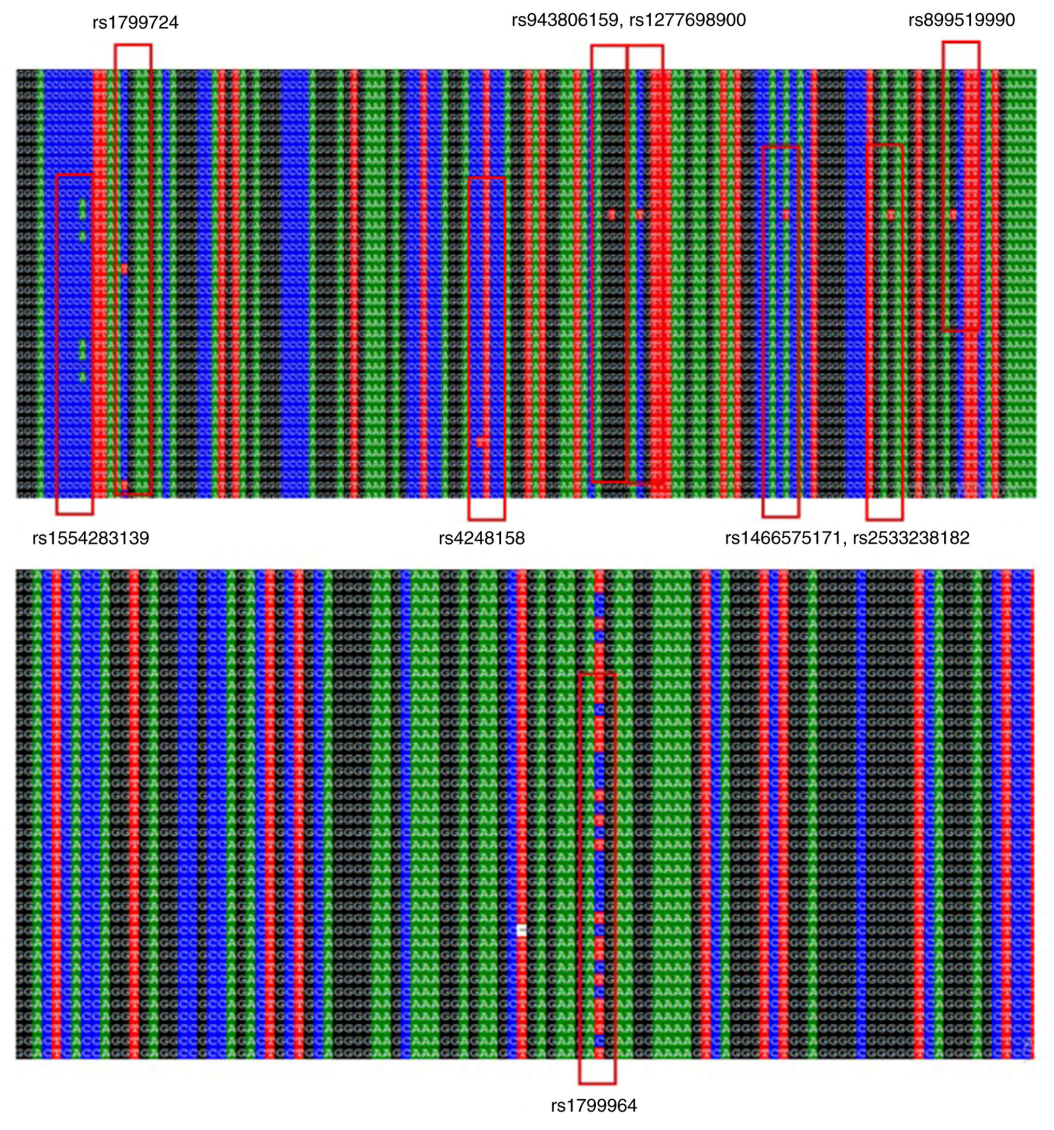
*TNF-α* promoter mutations detected in Epstein-Barr virus-positive samples. Red boxes indicate the locations of point mutations. Different rows correspond to different samples.

TNF-α promoter mutations were most commonly identified at the following SNPs in EBV-positive vs. EBV-negative individuals: rs1452146766, TTTT>TTTTT (→T), 66.7 vs. 9.1%; rs1799964, T>C (-1031 promoter), 58.3 vs. 33.3%; rs1554283139, CCCCCCC>CCCCCAC, 25 vs. 0.0%; rs924800313, C>A, 11.1 vs. 0.0%; rs1799724, C>T, 8.3 vs. 0.0%; and rs1771099055, CCCCC/CCCCCC, 7.7 vs. 0.0% (Table III; Figs. 4 and Fig. S1, Fig. S2, Fig. S3, Fig. S4, Fig. S5, Fig. S6, Fig. S7, Fig. S8, Fig. S9, Fig. S10, Fig. S11 and Fig. S12). Detection of the *TNF-α* promoter (size, 1,102 bp) is shown in Fig. S13.

### Conventional PCR

A significant association was also observed between the *TNF-α* mutation (TC) and mouth ulcers in individuals aged 3-50 years. The TC genotype was more common in individuals with mouth ulcers (42.4%) compared with the CC genotype (27.6%) (P=0.043; OR, 1.933; 95% CI, 1.016-3.678; Table IV). The expected PCR product sizes were 444 and 316 bp for the TT genotype, 444, 316 and 174 bp for the TC genotype, and 444 and 174 bp for the CC genotype (38) (Figs. S14 and S15).

## Discussion

The present study demonstrated that EBV reactivation varied across age groups, with a high EBV prevalence observed in individuals aged 11-20 and 21-30 years compared with individuals in other age groups. A previous report has shown that EBV variants, such as the 30-bp deletion *LMP-1* variant, are associated with malignant transformation (20). According to the World Health Organization, the key genes used for investigating EBV infection include *Bacillus amyloliquefaciens* (strain H) W repeat type II restriction enzyme, *EBNA-1* and *EBER* (45,46).

The present study identified several risk factors for EBV reactivation in oral buccal cells, including age, tobacco use and alcohol consumption; factors that are also linked to oral cancer risk in Thailand (47). Tobacco smoking and alcohol consumption were more prevalent among men, whereas betel nut chewing was more commonly observed among women in the present study, which was consistent with a previous study (48). In a previous study, significant associations were observed between oral cancer and tobacco smoking (OR, 4.47; 95% CI, 2.00-9.99), alcohol consumption in women (OR, 4.16; 95% CI, 1.70-10.69) and betel nut chewing (OR, 9.01; 95% CI, 3.83-21.22) (49), all of which exhibited dose-response effects. Smoking is relatively uncommon among Thai women; however, betel nut chewing remains widespread, especially among older women (49). The univariate analysis revealed an association between *LMP-1* status and sexual activity. These findings suggested that changes in traditional oral habits, such as reduced betel nut chewing and use of traditional cigars, may have contributed to the decline in oral cancer rates among both men and women in Thailand (49).

The findings of the present study regarding the association between *TNF-α* mutation and EBV status also reinforced findings from previous meta-analyses that highlighted the impact of the *TNF-α* gene on OSCC and oral potentially malignant disorders. *TNF-α* position -308 mutation has been associated with increased oral cancer risk (50,51). TNF-α levels in both saliva and serum are being explored as potential biomarkers for early OSCC detection, tumor staging, differentiation and prognosis. However, TNF-α levels are also influenced by general inflammation and common oral diseases, thus complicating their interpretation (51). In the oral cavity, TNF-α is modulated by both the oral microbiome and periodontal diseases (51). One study found that TNF-α levels in patients with OSCC (28.9±14.6 pg/ml) were significantly higher than those in patients with oral premalignant lesions (10.5±7.4 pg/ml) and healthy controls (3.0±1.0 pg/ml) (P<0.01) (52). Another study reported salivary TNF-α levels of 27.75±30.94 pg/ml in patients with OSCC, compared with 8.6±7.27 pg/ml in controls (53).

The *TNF-α* (-1031 T/C) SNP has been associated with severe adult periodontitis in the Japanese population (54). Although the C allele is linked to higher TNF-α cytokine levels than the T allele, the difference is not statistically significant due to concurrent mutations in other regions (55). In the present study, EBV reactivation was associated with the *TNF-α* (rs1799964; -1031 T/C) mutation. Consistent with earlier findings (44), the present study also demonstrated that this mutation was linked to the presence of mouth ulcers in oral samples from the Thai population. In other populations, such as in Iran, the same SNP has been associated with both the risk and severity of oral lichen planus (44). Future research should examine the annual frequency of mouth ulcers and their potential link to oral cancer. The complex relationship among EBV, the *TNF-α* (rs1799964; -1031 T/C) mutation and mouth ulcers warrants further investigation. These factors (such as mouth ulcers, alcohol consumption or smoking), along with EBV DNA and TNF-α cytokine levels, may ultimately be useful for oral cancer screening and head and neck cancer diagnostics. TNF-α (-1031 T/C) variants and mouth ulcers appeared to be associated in individuals aged 3-50 years. This indicates that the findings of the present study are exploratory, and thus, require further validation.

In a previous study, oral cancer was one of the most common forms of head and neck cancer, ranking as the sixth most common cancer among Thai men and being among the leading cancers in Thailand based on the mean annual ASR of the 2019-2021 period (5-11). Trends indicate an increasing incidence of oral cavity cancer in men (5-11).

In another study, the youngest male patient with oral cancer was 15 years old, while the youngest female patient was 18 years old (44,47). The median age among younger patients was 33.5 years (interquartile range, 42.5-24.5) (44,47). Nasopharyngeal cancer has been reported in male patients as young as 10-19 years old, with the incidence markedly increasing after the age of 50 years. Oropharyngeal and hypopharyngeal cancers in female patients have also been found, starting in the 40-44-year-old age group. Nasopharyngeal cancer in male patients often appears earlier, between the ages of 10 and 19 years, while oropharyngeal and hypopharyngeal cancers are more commonly observed in the 30-39-year-old age group (56). The cause of rising cancer rates among younger individuals remains unclear, and it continues to be debated whether this stems from genetic or environmental factors (15,16). The high prevalence of EBV in individuals aged 11-30 years should be closely examined as a potential risk factor.

A review of previous studies on EBV status in head and neck cancers in the Thai population published between 1991 and 2025 was conducted by including studies employing PCR/qPCR methods targeting the *Bam*HI N leftward frame 1, *LMP-1* and *EBNA-1* genes, as well as those involving the serological detection of anti-EBV IgG (20,23,57-76). The prevalence of EBV in normal tissue samples ranged from 5 to 33.7%, while in carcinoma samples, it ranged from 21 to 98% based on PCR/qPCR results. In normal blood samples, EBV prevalence ranged from 0 to 7.26% based on PCR/qPCR results. Using anti-EBV IgG serology, EBV prevalence ranged from 3.1 to 97.27% in normal blood samples and reached ≤86.5% in carcinoma cases. Examination of existing publications from 1991 to 2025 on head and neck cancers in the Thai population (20,23,55-76) showed that most studies focused on individuals aged 40-67 years, showing an ASR range of 0.1-14.68 among female patients and 0.6-15.7 among male patients. From 2001 to 2021, the prevalence and ASR of oral cavity and oropharyngeal cancers in Thailand increased, particularly among male patients (20,23,57-76).

The ASRs of head and neck cancers are generally lower in younger populations (<40 years old) compared with those in older adults. In young adults, the ASR of thyroid cancer was 14.4 per 100,000, while that of oral cavity cancer was 1-2 per 100,000 new cancer cases worldwide in 2022 (77,78). To the best of our knowledge, there are no studies on the ASR in this group in Thailand; only medical opinions are available, with no research to support existing opinions.

A limitation of the present study is that qPCR was not used to confirm cancer diagnoses. In another study, nucleic acid sequence-based amplification or reverse transcription-PCR were used for the detection of *EBNA-1, EBNA-2, LMP-2A* and *BZLF-1* (79), and immunohistochemistry was used for the detection of EBER (pathology confirmed), LMP-1, EBNA1, EBNA2, LMP2A and BZLF-1(77). *In situ* hybridization was used for the detection of EBER-1 and EBER-2 or EBV DNA. *EBNA-1*, *LMP-1*, *LMP-2A* and *BZLF-1* were most commonly used to detect EBV via qPCR (78,79). Improvements in quantitative amplification technology are stimulating a resurgence of interest in amplification strategies for detecting EBV in patient samples (80).

In conclusion, the alignment of EBV reactivation, age (11-30 years) and associated behavioral risk factors (alcohol consumption, smoking and sexual activity) strongly mirrors the risk profile for head and neck cancers. An association was found between *TNF-α* promoter mutations [such as rs1799964 (-1031 T/C)] and mouth ulcers or EBV. Future research should focus on integrating EBV DNA, *TNF-α* gene mutations and cytokine levels into early screening strategies for individuals with a high risk of OSCC.

## Supplementary Material

*TNF-α* promoter mutation (rs1799724) detected by sequencing.

*TNF-α* promoter mutation (rs4248158) detected by sequencing.

*TNF-α* promoter mutation (rs943806159 and rs1277698900) detected by sequencing.

*TNF-α* promoter mutation (rs1771099055) detected by sequencing.

*TNF-α* promoter mutation (rs1466575171) detected by sequencing.

*TNF-α* promoter mutation (rs2533238182) detected by sequencing.

*TNF-α* promoter mutation (rs899519990) detected by sequencing.

*TNF-α* promoter mutation (rs1799964) detected by sequencing.

*TNF-α* promoter mutation (rs1800629) detected by sequencing.

*TNF-α* promoter mutation (rs924800313) detected by sequencing.

*TNF-α* promoter mutation (rs1452146766) detected by sequencing.

*TNF-α* promoter mutation (rs1554283139) detected by sequencing.

PCR detection of *TNF-α* promoter (size, 1,102 bp).

Example of *TNF-α* promoter (-1031; CC and TC) detected by PCR.

Example of *TNF-α* promoter (-1031; TT) detected by PCR.

Univariate analysis of positivity versus negativity of *EBNA-1*, *LMP-1* and both genes.

Percentage of exposure to risk factors of oral cancer across age groups.

## Figures and Tables

**Table I:** Epstein-Barr virus-status by age, sex and *TNF-α* genotype (rs1799964; -1031 T>C).

	*EBNA-1*			*LMP-1*			Both genes positive		
Variable	Positive, n (%)	Negative, n (%)	Total, n	Positive, n (%)	Negative, n (%)	Total, n	Positive, n (%)	Negative, n (%)	Total, n
Age, years									
3-10	56(56)	44(44)	100	32(32)	68(68)	100	12(12)	88(88)	100
11-20	118(36)	209(64)	327	190(68)	89(32)	279	87(27)	240(73)	327
21-30	58(31)	128(69)	186	93(63)	55(37)	148	41(21)	151(79)	192
31-40	43(43)	56(57)	99	38(39)	59(61)	97	20(20)	80(80)	100
41-50	14(16)	73(84)	87	31(36)	56(64)	87	8(9)	79(91)	87
51-90	61(35)	114(65)	175	74(43)	100(57)	174	28(16)	147(84)	175
Total	350(36)	624(64)	974	458(52)	427(48)	885	196(20)	785(80)	981
P-value	<0.001	<0.001	<0.001						
Sex									
Female	233(35)	442(65)	675	339(55)	276(45)	615	138(20)	543(80)	681
Male	117(39)	184(61)	301	119(44)	153(56)	272	58(19)	243(81)	301
Total	350(36)	626(64)	976	458(52)	429(48)	887	196(20)	786(80)	982
P-value	0.190			0.002			0.719		
Odds ratio (female vs. male; 95% CI)	0.829 (0.626-1.098)			1.579 (1.185-2.105)			1.065 (0.756-1.499)		
*TNF-α* genotype									
TC	134(40)	205(60)	339	141(42)	197(58)	338	59(17)	282(83)	341
CC	71(37)	121(63)	192	59(31)	132(69)	191	18(9)	175(91)	193
Total	205(39)	326(61)	531	200(38)	329(62)	529	77(14)	457(86)	534
P-value	0.562			0.014			0.012		
Odds ratio (TC vs. CC; 95% CI)	1.114 (0.773-1.605)			1.601 (1.100-2.331)			2.034 (1.161-3.563)		

P-values were calculated using the χ^2^ test. *EBNA-1*, Epstein-Barr nuclear antigen-1; *LMP-1*, latent membrane protein-1.

**Table II:** Association between lifestyle factors and Epstein-Barr virus status.

	*EBNA-1*			*LMP-1*			Both genes positive		
Variable	Positive, n (%)	Negative, n (%)	Total, n	Positive, n (%)	Negative, n (%)	Total, n	Positive, n (%)	Negative, n (%)	Total, n
Sex									
Male	99(33)	200(67)	299	113(45)^a^	136(55)	249	42(14)^a^	257(86)	299
Female	108(36)^a^	191(64)	299	84(31)	188(69)	274	34(11)	265(89)	299
Total	207(35)	391(65)	598	197(38)	324(62)	521	76(13)	522(87)	598
Age, years									
3-10	56(56)^a^	44(44)	100	32(32)	68(68)	100	12(12)	88(88)	100
11-20	44(35)	83(65)	127	36(30)	83(70)	119	13(10)	114(90)	127
21-30	14(19)	58(81)	72	27(73)^a^	10(27)	37	9(12)	63(88)	72
31-40	19(19)	81(81)	100	31(35)	57(65)	88	8(8)	92(92)	100
41-50	43(43)	56(57)	99	34(38)	56(62)	90	20(20)^a^	79(80)	99
51-90	31(31)	69(69)	100	37(43)	50(57)	87	14(14)	86(86)	100
Total	207(35)	391(65)	598	197(38)	324(62)	521	76(13)	522(87)	598
Children, n									
1	21(37)^a^	36(63)	57	19(37)	32(63)	51	10(18)^a^	47(82)	57
2	30(34)	58(66)	88	33(42)^a^	45(58)	78	13(15)	75(85)	88
3	5(31)	11(69)	16	3(25)	9(75)	12	2(12)	14(88)	16
4	0 (0)	3(100)	3	0 (0)	3(100)	3	0 (0)	3(100)	3
No children (0)	120(36)	214(64)	334	105(36)	185(64)	290	37(11)	297(89)	334
Total	176(35)	322(65)	498	160(37)	274(63)	434	62(12)	436(88)	498
Sexual partners, n									
1	52(33)	106(67)	158	52(38)	86(62)	138	24(15)^a^	134(85)	158
2	3(50)^a^	3(50)	6	1(17)	5(83)	6	0 (0)	6(100)	6
3	0 (0)	2(100)	2	1(50)^a^	1(50)	2	0 (0)	2(100)	2
No sexual partners (0)	121(36)	211(64)	332	106(37)	182(63)	288	38(11)	294(89)	332
Total	176(35)	322(65)	498	160(37)	274(63)	434	62(12)	436(88)	498
Sexual activity									
Yes	101(31)	223(69)	324	121(44)^a^	154(56)	275	47(15)^a^	277(85)	324
No	106(39)^a^	168(61)	274	76(31)	170(69)	246	29(10)	245(90)	274
Total	207(35)	391(65)	598	197(38)	324(62)	521	76(13)	522(87)	598
Congenital disease									
Yes	20(30)	46(70)	66	21(36)	37(64)	58	8(12)	58(88)	66
No	186(35)^a^	343(65)	529	175(38)^a^	285(62)	460	68(13)^a^	461(87)	529
Total	206(35)	389(65)	595	196(38)^a^	322(62)	518	76(13)	519(87)	595
Family history of cancer									
Yes	8(26)	23(74)	31	9(31)	20(69)	29	3(10)	28(90)	31
No	199(35)^a^	368(65)	567	188(38)^a^	304(62)	492	73(13)^a^	494(87)	567
Total	209(35)	391(65)	598	197(38)	324(62)	521	76(13)	522(87)	598
Betel nut chewing									
Yes	23(33)	46(67)	69	15(23)	51(77)	66	5(7)	64(93)	69
No	184(35)^a^	345(65)	529	182(40)^a^	273(60)	455	71(14)^a^	454(86)	525
Total	207(35)	391(65)	598	197(38)	324(62)	521	76(13)	518(87)	594
Mouth ulcer									
Yes	69(29)	171(71)	240	85(41)^a^	120(59)	205	30(12)	210(88)	240
No	138(39)^a^	220(61)	358	112(35)	204(65)	316	46(13)^a^	312(87)	358
Total	207(35)	391(65)	598	197(38)	324(62)	521	76(13)	522(87)	598
Alcohol consumption									
Yes	89(32)	190(68)	279	116(51)^a^	111(49)	227	45(16)^a^	234(84)	279
No	118(37)^a^	201(63)	319	81(28)	213(72)	294	31(10)	288(90)	319
Total	207(35)	391(65)	598	197(38)	324(62)	521	76(13)	522(87)	598
Smoking									
Yes	29(36)^a^	51(64)	80	34(49)^a^	36(51)	70	19(24)^a^	61(76)	80
No	178(34)	340(66)	518	163(36)	288(64)	451	57(11)	461(89)	518
Total	207(35)	391(65)	598	197(38)	324(62)	521	76(13)	522(87)	598
Hot tea consumption									
Yes	48(31)	107(69)	155	62(48)^a^	67(52)	129	26(17)^a^	129(83)	155
No	159(36)^a^	284(64)	443	135(34)	257(66)	392	50(11)	393(89)	443
Total	207(35)	391(65)	598	197(38)	324(62)	521	76(13)	522(87)	598
Coffee consumption									
Yes	36(35)^a^	68(65)	104	38(40)^a^	58(60)	96	16(15)^a^	88(85)	104
No	171(35)^a^	323(65)	494	159(37)	266(63)	425	60(12)	434(88)	494
Total	207(35)^a^	391(65)	598	197(38)	324(62)	521	76(13)	522(87)	598
Soft drink consumption									
Yes	91(47)^a^	101(53)	192	61(32)	127(68)	188	24(17)^a^	116(83)	140
No	116(29)	290(71)	406	136(41)^a^	197(59)	333	52(13)	354(87)	406
Total	207(35)	391(65)	598	197(38)	324(62)	521	76(14)	470(86)	546
Herbal water consumption									
Yes	5(26)	14(74)	19	7(64)^a^	4(36)	11	3(16)^a^	16(84)	19
No	202(35)^a^	377(65)	579	190(61)	123(39)	313	73(13)	506(87)	579
Total	207(35)^a^	391(65)	598	197(61)	127(39)	324	76(13)	522(87)	598^a^

^a^Highest prevalence. *EBNA-1*, Epstein-Barr nuclear antigen-1; *LMP-1*, latent membrane protein-1.

**Table III:** Prevalence of *TNF-α* promoter SNPs by EBV status.

		EBV		
RS no.	SNP	Positive, n (%)	Negative, n (%)	Sequence from NCBI (5'-3')
rs1800629	G>A (-308)	0/3 (0.0)	2/8 (25.0)	GGGGCATG [G/A] GGACGGGGTT
rs1452146766	TTTT>TTTTT (->T)	2/3 (66.7)	1/11 (9.1)	GG [-/T] GAGGGGCATGGGG
rs1799964	T>C (-1031)	14/24 (58.3)	5/15 (33.3)	GAAGA [T/C] GAAGGAAAA
rs1799724	C>T	2/24 (8.3)	0/15 (0.0)	CCCCCCCTTAA [C/T] GAAGACAGGG
rs1554283139	CCCCCCC>CCCCCAC	6/24 (25.0)	0/15 (0.0)	CCCCC [-/A] CTTAACGAAG
rs4248158	C>T	1/24 (4.2)	0/15 (0.0)	CCTCCAGGAC [C/T] TCCAGGTATGG
rs943806159	G>C (found G>T)	1/24 (4.2)	0/15 (0.0)	ATACAG [G/C] GGACGTTTAAGAA
rs1277698900	C>T	1/24 (4.2)	0/15 (0.0)	GGGGA [C/T] GTTTAAGAA
rs1466575171	A>T	1/24 (4.2)	0/1 (0.0)	GGCCAC [A/G] CACTGGGGCCC
rs2533238182	G/- (found G>T)	1/24 (4.2)	0/15 (0.0)	GGGGCCCTGA [G/-] AAGTG
rs899519990	G>T	1/24 (4.2)	0/15 (0.0)	GAAGTGAGA [G/T] CTTCATGAAAAAAAT
rs1771099055	CCCCC/CCCCCC	1/13 (7.7)	0/14 (0.0)	CAAG [-/CC] AGCTCCTTCTCCCC
rs924800313	C>A	1/9 (11.1)	0/15 (0.0)	TTTTCCCTCCAAC [C/A/G] CCGTTTT

The point mutation is indicated in square brackets. EBV, Epstein-Barr virus; NCBI, National Center for Biotechnology Information; RS, reference SNP cluster ID.

**Table IV:** *TNF-α* (-1031; TC) mutation and presence of mouth ulcers.

*TNF-α* (-1031)	Mouth ulcers, n (%)	No mouth ulcers, n (%)	Total, n	P-value (odds ratio; 95% CI)
TC	81 (42.4)	110 (57.6)	191	0.043 (1.933; 1.016-3.678)
CC	16 (27.6)	42 (72.4)	58	
Total	97	152	249	

Data from individuals aged 3-50 years are included in the table. The P-value was calculated using the χ^2^ test.

## Data Availability

The data generated in the present study are included in the figures and/or tables of this article.
