# Supplementary material for: Investigation of genetic and lifestyle risk factors associated with Epstein-Barr virus reactivation in the Thai population
Source: Biomed Rep. 2026 Feb 20;24(4):50. doi: 10.3892/br.2026.2123 (PMC12976857; doi:10.3892/br.2026.2123)

Figure S1. *TNF-α* promoter mutation (rs1799724) detected by sequencing.

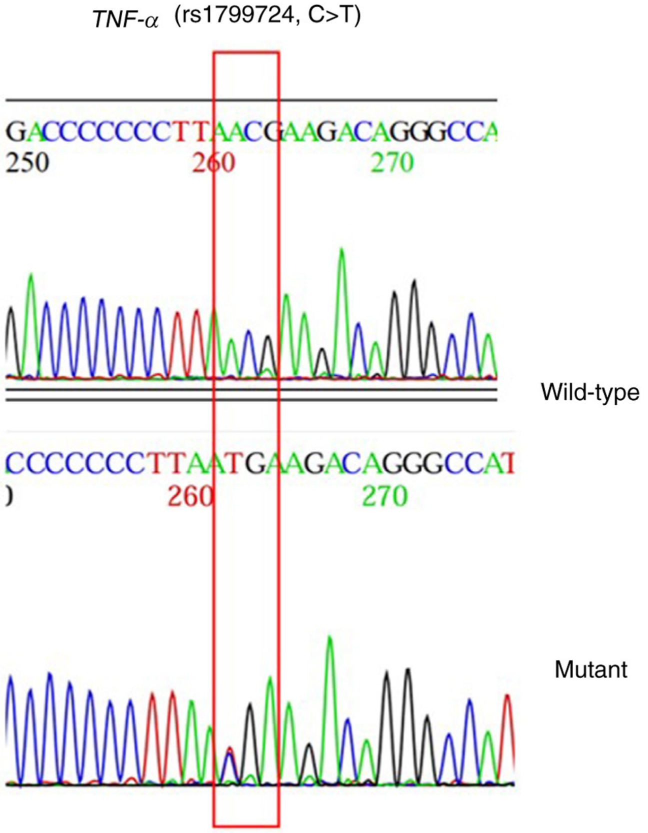

Figure S2. *TNF-α* promoter mutation (rs4248158) detected by sequencing.

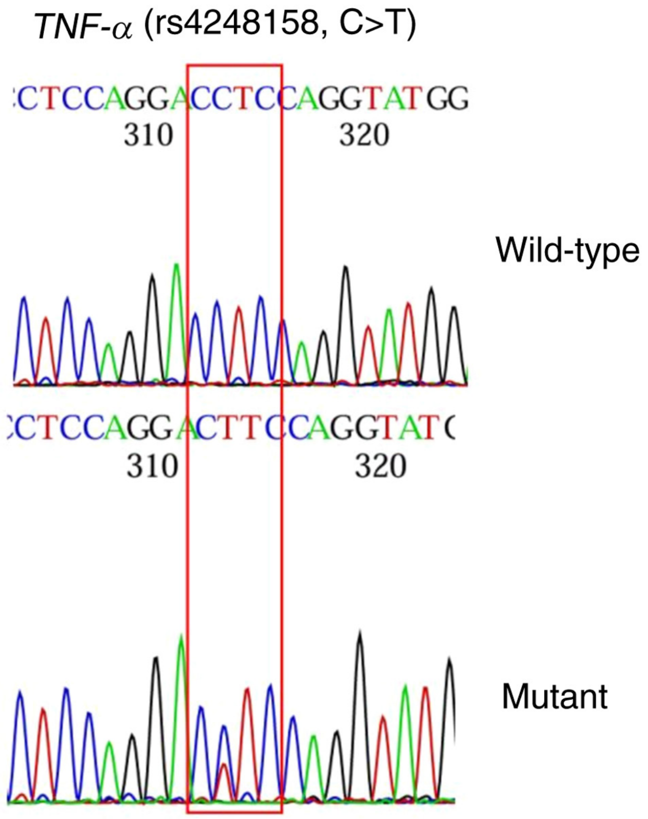

Figure S3. *TNF-α* promoter mutation (rs943806159 and rs1277698900) detected by sequencing.

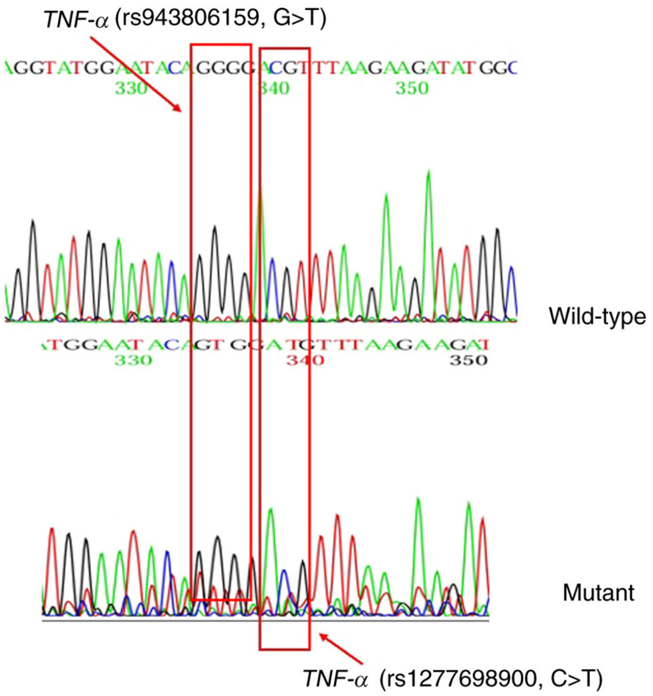

Figure S4. *TNF-α* promoter mutation (rs1771099055) detected by sequencing.

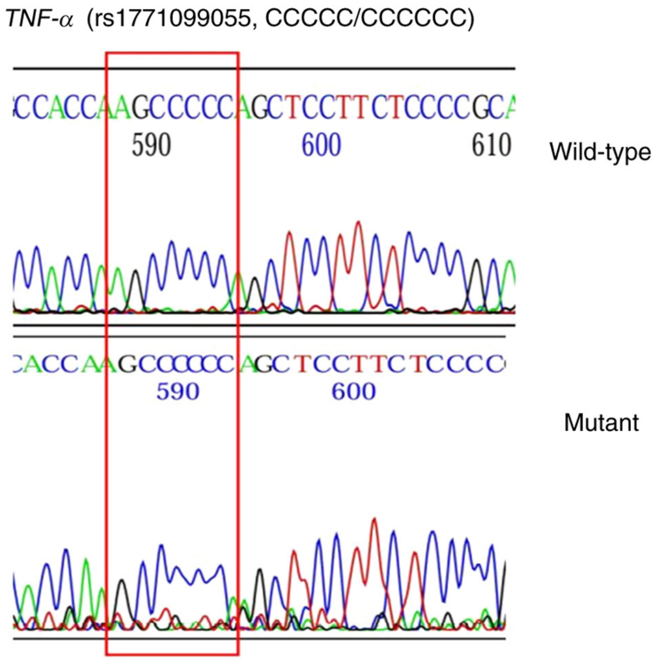

Figure S5. *TNF-α* promoter mutation (rs1466575171) detected by sequencing.

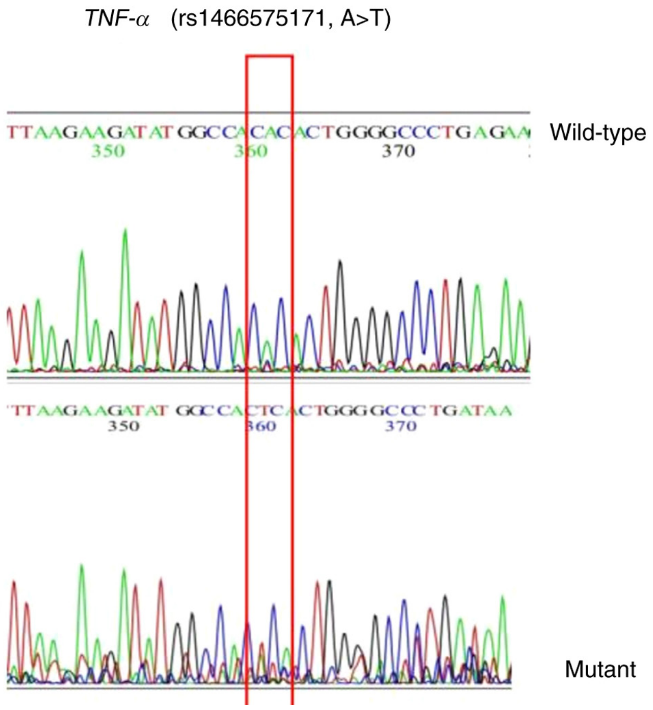

Figure S6. *TNF-α* promoter mutation (rs2533238182) detected by sequencing.

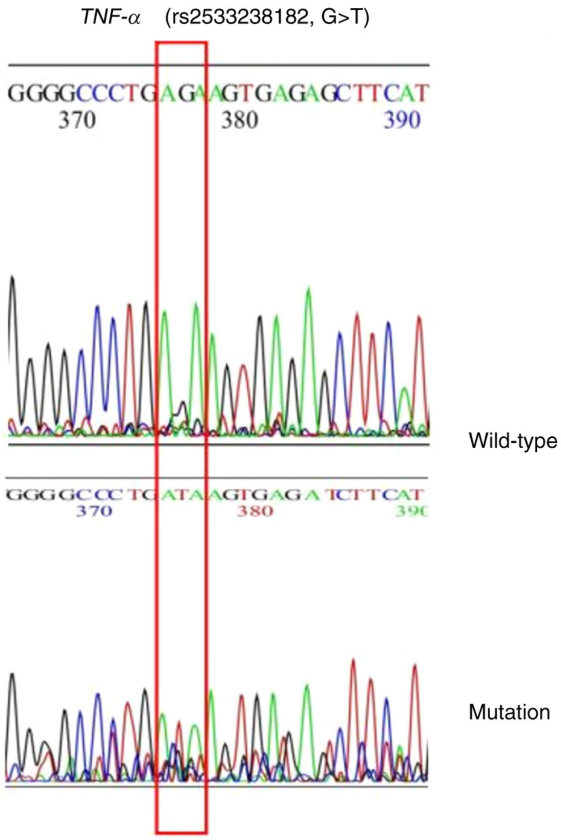

Figure S7. *TNF-α* promoter mutation (rs899519990) detected by sequencing.

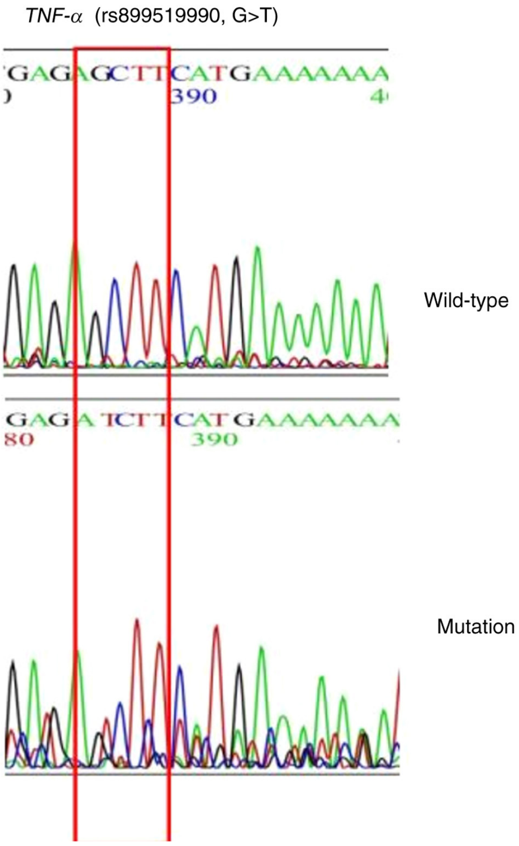

Figure S8. *TNF-α* promoter mutation (rs1799964) detected by sequencing.

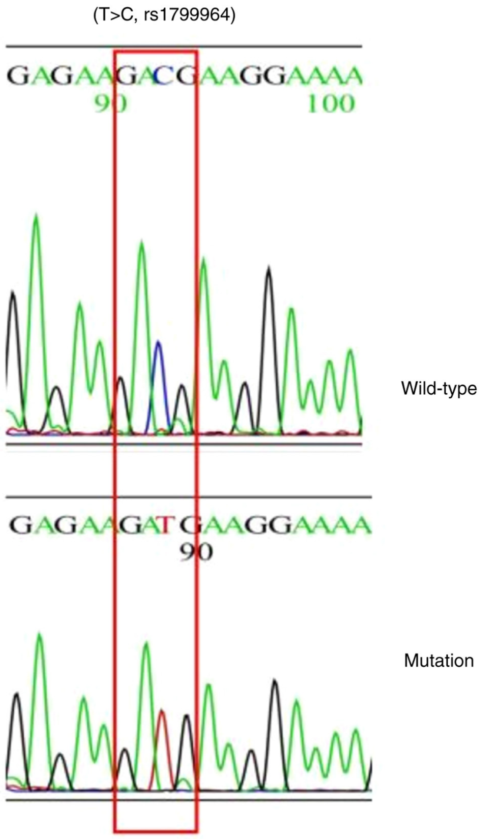

Figure S9. *TNF-α* promoter mutation (rs1800629) detected by sequencing.

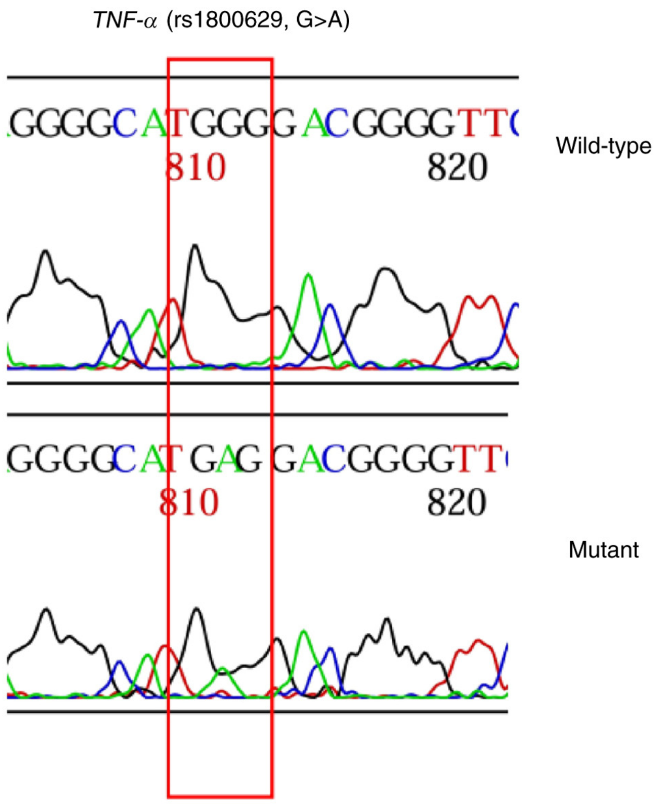

Figure S10. *TNF-α* promoter mutation (rs924800313) detected by sequencing.

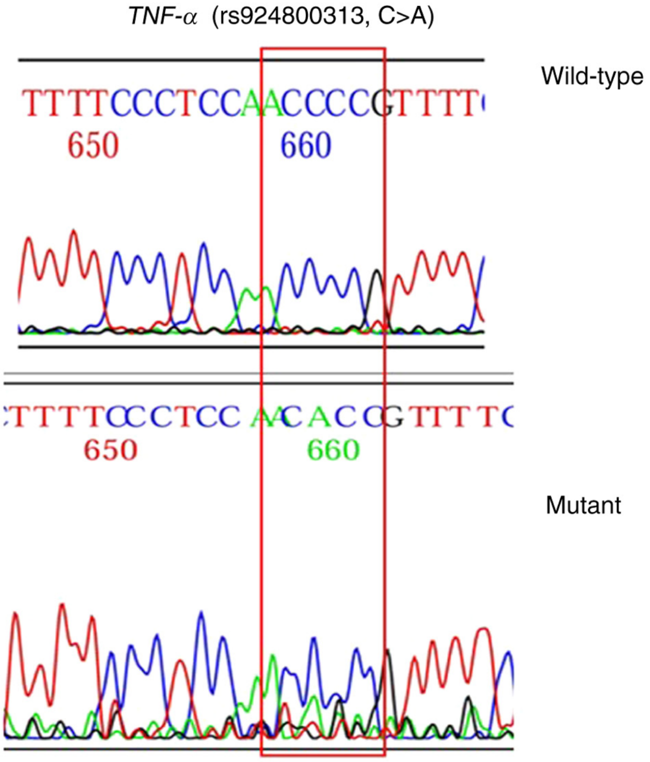

Figure S11. *TNF-α* promoter mutation (rs1452146766) detected by sequencing.

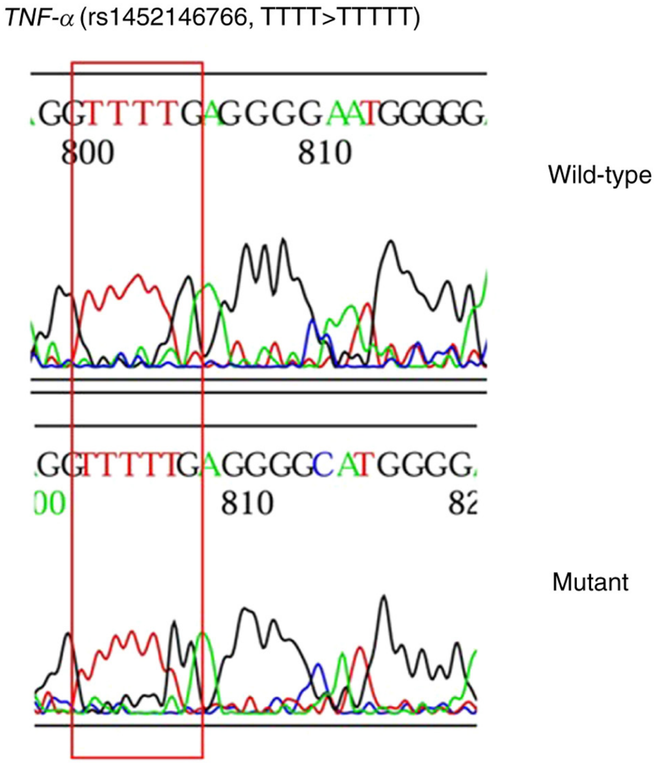

Figure S12. *TNF-α* promoter mutation (rs1554283139) detected by sequencing.

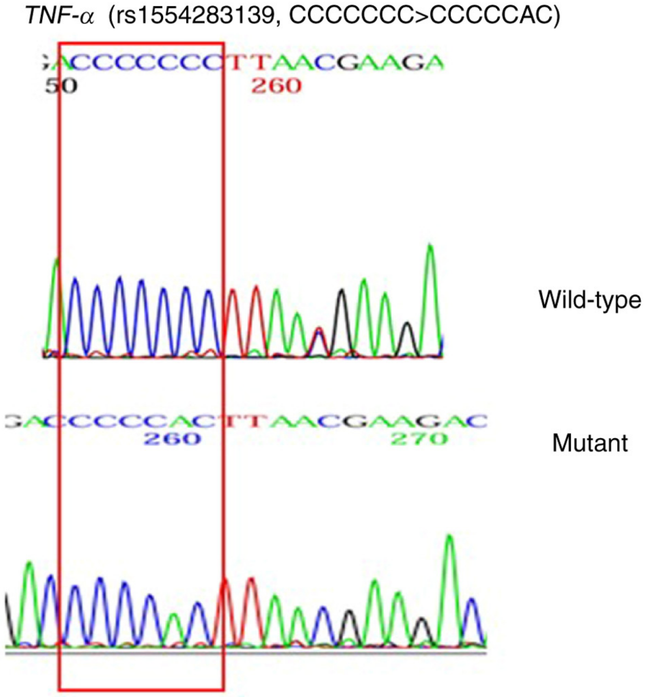

Figure S13. PCR detection of *TNF-α* promoter (size, 1,102 bp).

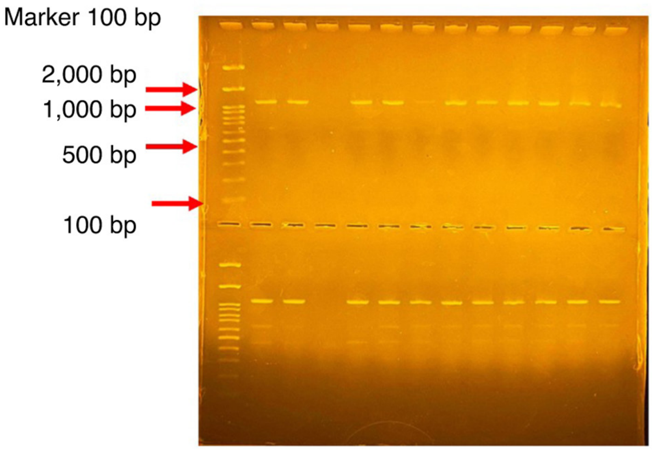

Figure S14. Example of *TNF-α* promoter (-1031; CC and TC) detected by PCR.

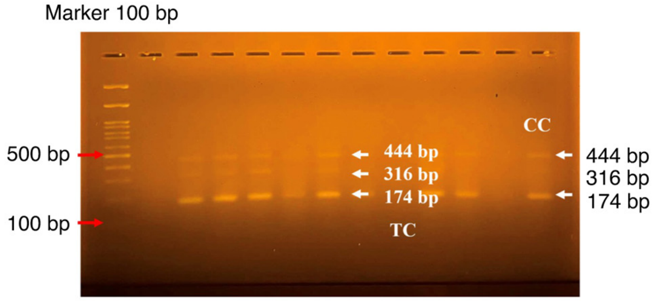

Figure S15. Example of *TNF-α* promoter (-1031; TT) detected by PCR.

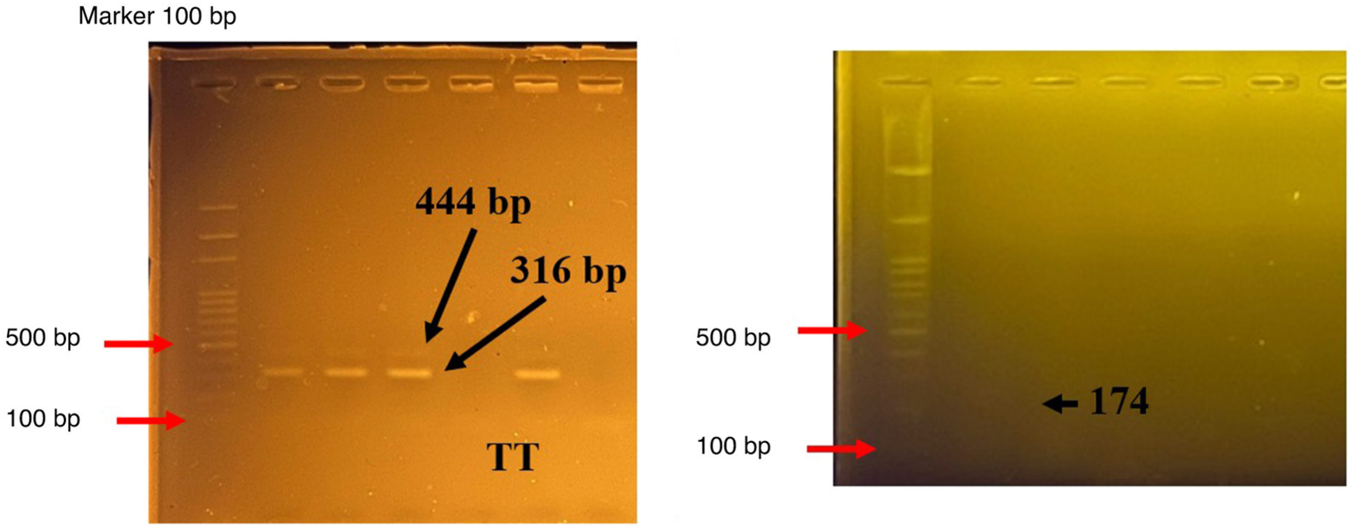

Supplement: TNF-α promoter mutation (rs1799724) detected by sequencing. [file Supplementary_Data1.pdf]
