# Supplementary material for: Investigation of genetic and lifestyle risk factors associated with Epstein-Barr virus reactivation in the Thai population
Source: Biomed Rep. 2026 Feb 20;24(4):50. doi: 10.3892/br.2026.2123 (PMC12976857; doi:10.3892/br.2026.2123)
Supplement: Univariate analysis of positivity versus negativity of EBNA-1, LMP-1 and both genes. [file Supplementary_Data2.pdf]

**Table SI.** Univariate analysis of positivity versus negativity of *EBNA-1*, *LMP-1* and both genes.

| A, *EBNA-1* status | | | | |
|---|---|---|---|---|
| **Risk factors** | **P-value** | **Odds ratio** | **95% CI lower ADR** | **95% CI higher ADR** |
| Mouth ulcer | 0.014 | 0.952 | 0.916 | 0.990 |
| Alcohol consumption | 0.192 | 0.975 | 0.939 | 1.031 |
| Sexual intercourse | 0.055 | 0.964 | 0.928 | 1.001 |
| Soft drink consumption | <0.001 | 1.094 | 1.052 | 1.139 |
| Age group 3-10 years | 0.001 | 2.401 | 1.402 | 4.112 |
| Age group 11-20 years | <0.001 | 5.273 | 2.606 | 10.667 |
| Age group 21-30 years | <0.001 | 5.426 | 2.870 | 10.257 |
| Age group 31-40 years | 0.077 | 1.658 | 0.947 | 2.902 |
| Age group 41-50 years | <0.001 | 2.833 | 1.583 | 5.056 |
| **B, *LMP-1* status** | | | | |
| **Risk factors** | **P-value** | **Odds ratio** | **95% CI lower ADR** | **95% CI higher ADR** |
| Sex | <0.001 | 1.860 | 1.300 | 2.661 |
| Betel nut chewing | 0.008 | 0.441 | 0.241 | 0.808 |
| Alcohol consumption | <0.001 | 2.748 | 1.908 | 3.958 |
| Smoking status | 0.048 | 1.669 | 1.006 | 2.769 |
| Sexual intercourse | 0.002 | 1.758 | 1.226 | 2.520 |
| Hot tea consumption | 0.006 | 1.762 | 1.177 | 2.637 |
| Soft drink consumption | 0.058 | 0.696 | 0.478 | 1.013 |
| Age group 11-20 years | <0.001 | 0.174 | 0.075 | 0.403 |
| Age group 41-50 years | 0.138 | 0.636 | 0.350 | 1.156 |
| **C, Both genes** | | | | |
| **Risk factors** | **P-value** | **Odds ratio** | **95% CI lower ADR** | **95% CI higher ADR** |
| 2 sexual partners | 0.245 | 1.386 | 0.799 | 2.403 |
| 4 children | 0.169 | 1.708 | 0.796 | 3.664 |
| Betel nut chewing | 0.155 | 0.504 | 0.196 | 1.295 |
| Alcohol consumption | 0.020 | 1.787 | 1.096 | 2.913 |
| Smoking status | 0.002 | 2.519 | 1.405 | 4.517 |
| Sexual intercourse | 0.153 | 1.433 | 0.875 | 2.349 |
| Hot tea consumption | 0.079 | 1.583 | 0.948 | 2.549 |
| Coffee consumption | 0.369 | 1.315 | 0.724 | 2.390 |
| Age group 31-40 years | 0.674 | 0.838 | 0.367 | 1.914 |

*EBNA-1*, Epstein-Barr nuclear antigen-1; *LMP-1*, latent membrane protein-1; ADR, average daily rate.

**Table SII.** Percentage of exposure to risk factors of oral cancer across age groups.

| Risk factor | 3-10 years | 11-20 years | 21-30 years | 31-40 years | 41-50 years | 51-90 years |
|---|---|---|---|---|---|---|
| Sex (male) | 59 | 57 | 100 | 59 | 27 | 36 |
| *EBNA-1* positive | 56 | 34 | 19 | 19 | 43 | 31 |
| *LMP-1* positive | 32 | 33 | 73 | 35 | 38 | 43 |
| Both *EBNA-1* and *LMP-1* positive | 12 | 10 | 13 | 8 | 20 | 14 |
| >1 children | 0 | 0 | 0 | 83 | 53 | 0 |
| >1 sexual partner | 0 | 0 | 0 | 0 | 1 | 0 |
| Sexual activity | 0 | 11 | 54 | 90 | 87 | 95 |
| Congenital diease | 2 | 4 | 0 | 6 | 5 | 38 |
| Family history of cancer | 3 | 0 | 0 | 9 | 7 | 12 |
| Betel nut chewing | 1 | 39 | 0 | 3 | 15 | 0 |
| Mouth ulcers | 22 | 55 | 68 | 48 | 13 | 38 |
| Alcohol consumption | 0 | 2 | 69 | 79 | 59 | 61 |
| Smoking | 0 | 2 | 18 | 16 | 21 | 28 |
| Tea consumption | 17 | 11 | 47 | 14 | 24 | 62 |
| Coffee consumption | 0 | 0 | 0 | 44 | 61 | 0 |
| Soft drink consumption | 99 | 50 | 0 | 43 | 0 | 0 |
| Herbal tea consumption | 0 | 0 | 0 | 19 | 0 | 0 |

*EBNA-1*, Epstein-Barr nuclear antigen-1; *LMP-1*, latent membrane protein-1.
